# The role of older women in contesting norms associated with female genital mutilation/cutting in Senegambia: A factorial focus group analysis

**DOI:** 10.1371/journal.pone.0199217

**Published:** 2018-07-25

**Authors:** Bettina Shell-Duncan, Amadou Moreau, Katherine Wander, Sarah Smith

**Affiliations:** 1 University of Washington, Seattle, Washington, United States of America; 2 Global Research and Advocacy Group, Dakar, Senegal; 3 University of Binghamton, Binghamton, New York, United States of America; Public Library of Science, UNITED KINGDOM

## Abstract

Social norms theory has become prominent framework for understanding the perpetuation of female genital mutilation/cutting (FGM/C), and has influenced the design of interventions aimed at stopping the practice. Theoretical advances draw attention to the fact that FGM/C is often upheld by multiple interconnected norms that may vary and shift over time, offering a potential resource for social transformation. Analyzing focus group data from Senegambian women, the questions we explore are: What are the constellation of norms associated with FGM/C? When are existing practices and norms being contested, and how does this reflect prevailing structures of power and authority? Our research identifies four overarching themes: 1) pressure to conform with FGM/C arising from sanctions such as ostracization, and moral norms linked to the embodiment of virtue; 2) upholding tradition as a means of venerating ancestors; 3) upholding social hierarchy by displaying respect for elders; and 4) shifting beliefs about the healthful vs. harmful nature of FGM/C. While strong value is placed on upholding tradition, there is also an appreciation that elements of tradition must be revised to meet fluctuating realities, including the novel threat of HIV infection. Moreover, older women are uniquely positioned to realize the dual goal of honoring tradition while negotiating change. Rather than resisting change, we find that some older women express an openness to reassessing norms and practices as they seek solutions to maintaining the physical well-being, moral integrity and cultural identity of girls in their families. Moreover, given the authority of older women over younger women, they also have power to negotiate change. By recognizing older women as potential change leaders, and drawing on variability and fluidity in social norms, it may be increasingly possible to design interventions that will shape possibilities for action and accelerate abandonment of FGM/C without undermining the cultural value of tradition.

## Introduction and background

Social norms theory has become a prominent framework for understanding health-related behaviors, and in recent years has been influential in the design of interventions aimed at generating social change. Norms perspectives have been used to address a wide range of issues such as eating habits [1], alcohol consumption [2], safer sex and sexual assault prevention [3], but particularly well-known application has been to the practice of female genital mutilation/cutting (FGM/C) [4]. The terms female genital cutting, female genital mutilation, the hybrid female genital mutilation/cutting and female circumcision are euphemistic terms used to describe a broad range of practices involving the alteration of the external female genitalia for non-therapeutic reasons. In a model first developed by political scientist Gerald Mackie [5], it was posited that FGM/C is a social norm that spread and became locked in place by interdependent expectations regarding marriageability. With the subsequent empirical investigations, as well as a growth in scientific literature on social norms, expanded views on FGM/C have been two-fold. First, they emphasize that FGM/C may be held in place not only by norms related to marriageability, but also by a wide range of norms and associated meanings that may center on concerns including ethnic identity, adolescent rites of passage, religion, honor, modesty and sexual restraint, aesthetics and hygiene [6]; it has been argued that it is useful to tease apart the constellation of norms and cultural values associated with FGM/C in order to gain insight into the ways the practice is promoted, enforced or upheld in various social settings [4, 7]. Second, theoretical perspectives of the role of social context in behavior change have become enriched by anthropological research that illuminates the way that historical and structural factors interact with inter-personal interactions and personal goals, and the way in which cultural variation provides the raw material for meeting these goals [8–11]. This work extends an earlier body of scholarship that has challenged understandings of culture as static and deterministic of behaviors. From this viewpoint, FGM/C had become labeled a “harmful traditional practice,” and adherents of the practice were understood to lack agency to interpret culture, evaluate options and make genuine choices. Culture was cast as a justification for following customs rooted in the past, posing a barrier to social transformation and reform efforts. An alternative perspective points to the diverse views and multiple meanings associated with FGM/C, and the dynamic process through which shared meanings and social knowledge are generated [7]. Emphasized are the fluidity of social norms and cultural values, and the contexts in which FGM/C becomes associated with meanings that can accrue, be lost, or altered, thereby influencing whether and how FGM/C is practiced. This understanding offers that social norms and associated meanings are neither static nor homogenous, but instead varied and constantly changing through processes of cultural borrowing and innovation [9–13]. Rather than being purely prescriptive, culture offers a range of options that define acceptable and unacceptable courses of action. An Na’Im and Hammond [10] offer the image of a “cultural tool kit” from which individuals construct strategies of action to meet their goals, while Hernlund [14] uses the metaphor of a winnowing basket to describe the process in which people actively negotiate which aspects of culture should be retained or discarded in light of current or shifting social realities. Recent theoretical advances in behavior change draw on these insights about social context, personal interactions and goals, and cultural variation to suggest strategies for guiding social transformation [10, 11]. Seeing culture as varied and fluid allows for the interpretation of cultural heterogenity as an indigenous “resource for change” rather than a repository for unchanging values and practices ([1]; p.9, see also [15, 16]).

Drawing on these theoretical perspectives, the goal of this research is to explore the social norms and dynamics that influence decision making regarding FGM/C in Senegal and The Gambia. The key questions we ask are: What are the constellation of norms and meaning associated with FGM/C? In what ways are they being shaped, upheld, resisted or modified in light of changing social circumstances? When and where are existing practices and norms being questioned, and how does this reflect prevailing structures of power and authority? In this study we explore these questions through the analysis of focus group data collected from Senegambian women.

### Factorial focus group methodology for the study of social norms and dynamics of change

The growing focus on social norms perspectives have generated interest in understanding how to identify social norms, along with the social contexts and cultural factors that shape them. Differing from legal norms, whose proscriptions and violations are explicitly codified, social norms are unwritten expectations regarding appropriate behaviors within particular social groups. They are held in place by beliefs about acceptable and unacceptable courses of action that can, to varying degrees, shape and constrain the choices made by community members, and are produced, transmitted and often enforced through social interactions [17]. As such, it is possible to shed light on norms through research approaches that includes social interactions as an object of inquiry.

Focus group interviews produce a distinctive means of generating data that can be particularly useful in exploratory investigations of social norms and the cultural meanings and values with which they are associated. Focus groups are structured, planned group interviews which are designed to elicit information on perceptions and attitudes, and to provide an opportunity to observe exchanges between participants, as well as their reactions to these exchanges. This generates useful information in several ways: What topics were raised in a particular group discussion? To what extent was there consensus or disagreement? What reactions were generated by divergent perspectives?

Unlike in-depth interviews, focus group methodologies are not well suited to explore the experiences and histories of individuals. For instance, questions about how an individual arrived at a decision as to whether, when or how their daughter should undergo FGM/C is a topic best suited for individual interviews. By contrast, focus groups are guided by semi-structured questions that serve as prompts for open-ended small group discussions. They allow participants to describe in detail understandings of cultural practices, preferences and values found within a community, along with perspectives on the circumstances and debates that frame certain issues. An important part of this process is to allow participant to raise issues and explanations they deem to be salient. For instance, while eliciting information on the meanings associated with FGM/C, the topics raised may vary across a series of focus groups, reflecting differences in the issues and concerns that participants hold most central. The dialogue among participants offer opportunities for critical reflection on deeper meanings, and may reveal divergent views that can spark debate or disagreement. The topics raised reflect the constellation norms and cultural meanings, and exchanges between participants shed light on the degree to which they have become expanded, altered or contested.

Participant observation, like focus group methods, affords an opportunity to examine social interactions that can shape cultural values and normative expectations. While participant observation bears the advantage of observing interactions in naturalistic settings, it can be difficult to gain access to settings in which a substantial set of interactions can be observed on the topic of interest [18]. Focus group methodologies provide an opportunity to observe a large set of interactions, and while they do not occur in naturalistic settings, this limitation is also a strength: they allow researchers to exercise control over group discussions, and to hold a series of discussions to generate information that can identify patterns across segments of a community.

One particularly powerful comparative approach to focus group methodologies involve factorial designs [18, 19]. When there is interest in comparing views across different subgroups, this approach involves holding discussions with separate groups, each homogenous in terms of having common *control characteristics*, but different in terms of as *break characteristics*–that is, characteristics that differentiate groups from each other [19]. Break characteristics are used as the basis for developing a factorial design. For instance, if one were to use one control characteristic, such as being female, along with two break characteristics, age (over 30 years of age vs. under 30 years of age) and location of residence (urban vs. rural), four focus group categories are defined: younger urban women, older urban women, younger rural women and older rural women. By using common guidelines among discussions among different subsets of individuals, it is possible to draw comparisons and look for patterns or variation in cultural meanings and values, and identify when and where norms have become contested or transformed. Hence, we have adopted this approach in our investigation of norms surrounding the practice of FGM/C.

### Study population

This research project was conducted at sites located in the West African nations of Senegal and The Gambia. Often these nations are referred to collectively as Senegambia, owing to the fact that they share much in common in terms of language, dominant religion (over 90% Muslim in both), subsistence activities, cultural practices, landscape, and climate. Geographically, Senegal is the western-most country on the African continent, extending from the Atlantic coast to the western edge of the Sahel. The tiny nation of The Gambia is a long thin strip of land that extends from the Atlantic coast inward 300 kilometers along the banks of the Gambia River, and is an enclave, bordered on three sides by Senegal, which nearly bisects the nation of Senegal. The national borders are a vestige of colonial rule: Senegal was formerly ruled by France, while the British controlled the Gambia River. Post-independence, each nation has remained an autonomous state, despite sharing a common historical and cultural heritage.

As the practice of FGM/C is most closely tied to ethnic affiliation, the overall prevalence rates for The Gambia and Senegal vary. In Senegal, where the largely non-practicing Wolof are the largest ethnic group, the national prevalence of FGM/C is 25% [20]. In The Gambia, where the ethnic majority are Mandinka who near universally practice FGM/C, the national prevalence is 75% [20]. Inter-ethnic marriage has, however, become increasingly common, including marriage between groups that do and do not practice FGM/C. As a consequence of ethnic mixture, the conceptualization of ethnic identity is complex and often situational, sparking debates about “customary practices” particularly in relation to issues such as FGM/C.

Type I (clitoridectomy) and Type II (excision of the clitoris and labia minora) are the most common forms of FGM/C in Senegambia, and a variant of Type III known as sealing (clitoridectomy, excision of the labia minora and usually the labia majora, and appositioning the cut labial edges to fuse and form a seal over the vulva; unlike infibulation common in the Horn of Africa, this practice does not involve stitching together the cut edges of the labia) is reported less frequently [21, 22]. In the past FGM/C was commonly performed on groups of girls as an adolescent coming-of-age ritual accompanied by lengthy seclusion in the bush where initiates were provided training on moral etiquette and “women’s secrets”. In recent decades this has changed, and it has become reportedly common for girls to be cut individually at home, with little or no training or celebration [14, 23]. Additionally, there has been tendency to perform FGM/C at younger and younger ages [24], although in a few groups, such as Tukulor families, FGM/C has long been practiced during infancy [25]. Survey data from our study sites have confirmed these trends [26]: daughters undergo FGM/C, on average, 4 years earlier than did their mothers, and are a third more likely to experience what Hernlund called “cutting without ritual” [24].

Changes in the way that FGM/C is carried out have unfolded against a backdrop of debates sparked by ongoing campaigns aimed at ending FGM/C. Senegal and The Gambia have experienced very different trajectories regarding strategies to eliminate FGM/C. Senegal is the original site of the massive Tostan grassroots project, which as of 2009 organized public declarations to abandon FGM/C in over 3,700 villages [27]. In an effort to build upon Tostan’s early successes, in 1999 Senegal adopted legislation criminalizing the FGM/C within the country. In The Gambia, by contrast, efforts to introduce legislative reform measures for FGM/C were strongly resisted until 2015, well after our data collection had concluded in 2008 [20]. The Tostan program began work in The Gambia in the Upper River Region during our study, and by June, 2009, 24 communities participated in public declarations to end FGM/C [27]. At the time of our study, however, Gambian participants in our research were unaware of these activities, although all were familiar with other campaigns that had been underway for decades in The Gambia. One of the clearest effects of anti-FGM campaigns in both countries has been to produce a widespread awareness that this “local practice” has become the object of intense scrutiny through media coverage of “FGM” [24, 26]. Senegambians are not only consumers of media that has labeled FGM a “harmful traditional practice” and a violation of women’s rights, but have also repeatedly become the subjects of such reports. As a result, many are acutely aware that their customs are at the heart of local, national and global debates between those who perceive a need to abolish FGM/ and those who seek to preserve a practice that is an integral part of their culture.

Focus group data were collected in 2005–06 as part of a three-year mixed methods study on behavior change with respect to FGM/C. Research was conducted in three distinct regions: 1) peri-urban communities surrounding the Gambian capital, Banjul; 2) The Gambian North Bank Division border area known as Baddibu, and 3) the Senegalese region directly across the border from Baddibu, falling in what was then Kaolack Division (now Fatick). These research sites do not provide nationally-representative samples, but were selected instead to capture variation in the practice of FGM/C and social dynamics influencing decision-making around FGM/C. Residents in each of these sites typically live in large multigenerational families characterized by patrilineal descent. Women, upon marriage, move from their natal home to their marital home and forge relationships with female family members involved in collective caretaking of children. Our earlier research from this project investigated whether FGM/C is linked to marriageability, a factor posited by social convention theory to be responsible for the spread and continuation of FGM/C [28]. Our analyses revealed that FGM/C is most often only indirectly related to marriageability via concerns over preserving virginity [29]. Most community members emphatically maintained that FGM/C was neither a prerequisite to marriage nor a means of securing a better marriage. Instead, FGM/C was found to be upheld by a peer convention. We proposed that FGM/C serves to signal to other circumcised women that a girl or woman has been trained to respect the authority of her circumcised elders and is therefore worthy of inclusion in their social network [29]. Thus, FGM/C upholds an intergenerational hierarchy of power amongst women. This finding motivates the design of this current study which seeks to illuminate the constellation of norms and meanings that serve to uphold the practice of FGM/C, and understand where and how these norms are being challenged or altered.

In creating the factorial design of this study, our control characteristics are being a woman age 18 or older who comes from a family with a history of practicing FGM/C (opening the possibility that women and their families may have recently abandoned the practice of FGM/C). Comparisons are made across two potentially important break characteristics: residence (urban Gambia vs. rural Gambia vs. rural Senegal) and age (younger vs. older women). We use this design to ask questions about some common assumptions regarding patterns of social transformation regarding FGM/C:

Are older women, who accrue power and exert their power over younger circumcised women, steadfast guardians of tradition and resistant to change regarding FGM/C?
Are urban Gambian women more highly exposed to and influenced by campaign messages and the transnational discourse opposing FGM/C than their rural counterparts?
In rural Senegal, where FGM/C is found among a minority of women and is banned by a national law, are women more likely to be influenced by non-cutting members of their society and prevailing social and legal norms opposed to FGM/C?

## Methods

### Study design and data collection

Ethical approval of this study was obtained from Gambia College, Brikama Campus, Université Cheikh Anta Diop in Dakar, University of Washington in Seattle, and the World Health Organization in Geneva. The study protocol was also approved by a community advisory board in The Gambia and by the WHO Office in Dakar. The community advisory board supervised the data collection, and organized validation seminars in both Banjul and Dakar prior to dissemination of research findings. Following protocols established previously in The Gambia, participants were read an informed consent statement in their language of choice (Mandinka or Wollof). They either signed or thumb-printed the statement. They were given a card with information for contacting the local WHO office or the University of Washington to voice any concerns regarding the study.

Focus group discussions (FGDs) were held among Senegambian women to identify social norms associated with FGM/C for different stakeholders: girls who themselves may have or will undergo FGM/C, women in the community, as well as fathers, husbands or other men in the community. The focus group guidelines were designed to elicit normative statements by prompting discussion on attitudes and opinions about FGM/C, as well as perceptions of behavioral patterns. The guidelines focused on four broad questions:

1. What are the advantages of FGM/C? (probing: For girls? For women? For fathers? For husbands?)
2. What are the disadvantages of FGM/C? (same probes),
3. What are the solutions?, and
4. What changes have you seen in the practice of FGM/C and how do you feel about these changes?

The questions and probes were designed to be sufficiently open-ended to allow discussants to raise topics and perspectives that they considered salient. We explored the degree to which norms are shared or contested by analyzing both positive and negative associations reported by respondents, and group members’ degree of consensus around these evaluations. A total of 15 FGDs were held, with 6–8 participants in each group. FGDs were convened by two trained Gambian fieldworkers who had been residing in the study communities for the previous year while conducting participant observation and in-depth interviews. Thus, the field workers were well known and trusted in the study communities, which facilitated recruitment for FGDs. One fieldworker moderated the discussion, while the other digitally recorded the session and also took notes to guide transcription of recordings. The fieldworkers were fluent in local languages, and permitted participants to conduct discussions in their language of choice (mostly Mankinka or Wollof). They were trained to address and attempt to mitigate well known problems in focus group discussions: there is a risk that one or two participants dominate a discussion, or that critical comments may silence members whose views are not shared by other discussion participants. Facilitators were trained to encourage open and even participation, and to create a supportive and respectful atmosphere that permitted open sharing and exploration of a range of opinions. Because some statements cannot be interpreted at face value, but instead require interpretation based on the context in which they are made, fieldworkers provided a descriptive debriefing following each session. Additionally, once the recordings were translated and transcribed, the fieldworkers added comments describing statements such as jokes, statements with double meaning, segments were respondents seemed reticent or uneasy, and any other relevant observations they could offer from “closeness” to the data that would aid in interpretation and analysis of transcripts.

### Data management and analysis

Transcripts were shared with the data analysis team, and reviewed for completeness and clarity. Once all queries were resolved, the transcripts were coded using ATLAS.ti. We used a grounded theory approach to analyzing the transcripts. Grounded theory is an iterative process that involves successively more focused rounds of coding text to identify themes or categories, writing memos to explore themes and identify relations among themes or categories, and linking themes to building analytical models [18, 30]. We began with a close reading and open coding of the transcripts, double-coding the first three transcripts. Members of the analysis team met to compare and reconcile coding strategies, and worked together to build a list of codes and sub-codes. The first round involved topic coding that labeled key topics; this was followed by a round of analytical coding that began to group topics into themes [31].

A summary document was created for each FGD, identifying the major themes raised, grouped along the lines of advantages or disadvantages of FGM/C. Notes were made regarding the emphasis placed on each theme (ranging from being a passing remark to a topic that participants deeply explored), as well as the degree of consensus, debate or disagreement that came about during the discussion. This process served as an analytic approach to discerning the degree to which normative statements and positive or negative associations were shared among participants, debated, or possibly actively contested during the discussion. As recommended by Knodel [19], we used a team approach to both creating data summaries and identification of themes in an effort to improve reliability of our analysis. The data analysis team members independently identified topics and patterns, and then met to discuss interpretation of emerging themes, and to identify “exemplar quotes”–direct quotes from the transcripts that illustrated a concept or theme [18]. The starting point for this analysis was assessing the degree with which stated representations and understandings of FGM/C were shared, contested, or contradicted (by individual respondents themselves or between participants), reflecting ambivalence by at last some members of the discussion group. A key element in this analysis was exploring the possibility that practices, meanings and values associated with FGM/C may not be fixed and shared among all participants. Instead, we explored the possibility of potentially shifting, and at times inconsistent or contested views on FGM/C.

The summaries for each focus group were used to create an “overview grid” following methods of Knodel [19], allowing comparisons along the lines of break characteristics (younger vs. older women, and location of residence: urban Gambia, rural Gambia, and rural Senegal). Basic themes were grouped into overarching themes, and a summary matrix was created for each overarching theme [32]. This matrix summarized the direction of consensus as follows: positive consensus among discussants (+), negative consensus (-), divergent views (+/-), or a theme not raised (blank). Analytic memos were created to describe themes that emerged through the analysis, exploring patterns and overarching organizing themes that formed the basis for the interpretation of the results. For more information on analytic memos and displays for qualitative data, see [18, 31, 32].

## Results

In the process of analysis, emergent themes were grouped into four main overarching themes related to: 1) pressure to conform, 2) tradition, 3) upholding social hierarchy, and 4) health. While the first theme, pressure to conform, reflect the ways in which social norms are enforced through sanctions or internalization of moral values, the remaining themes describes the intertwined norms, meanings and rationales for performing FGM/C, and the ways in which these notions are being upheld or disputed. Each overarching themes is summarized here in a matrix that displays where subthemes were raised, along with patterns in which they were supported or contested.

### “You *solemas* (uncircumcised girls), you will not follow us (hang out with us)”: The power of pressure to conform

The emergent themes related to the overarching theme of pressure to conform are summarized in Table 1. We find that pressure to conform arises from several sources: negative sanctions for uncut girls or women and their families experienced in the form of ostracization and peer pressure or judgment on proper parenting, and internalized moral norms regarding the link between being cut and embodying virtue.

**Table 1:** Summary matrix of themes related to the overarching theme of pressure to conform.

	Urban Gambia		Rural Gambia		Rural Senegal	
	Younger Women	Older Women	Younger Women	Older Women	Younger Women	Older Women
Ostracization of uncut women	+	+		+		
Peer pressure among girls	+		+			
Proper parenting	+	+	+	+		+
Moral virtue	+	+	+	+	+	+

+ positive consensus.

+/- divergent views.

-negative consensus.

Blank—theme not raised.

With the growing acceptability of inter-ethnic marriages, it has become increasingly common for uncut women to marry into families that carry the tradition of FGM/C, and the frequent intra-familial discord between cut and uncut women throws into sharp relief the ostracization of those women who have not undergone FGM/C. Uncut women from these FGM/C-discordant families commonly report not just disapproval from female kin in their marital homes and community, but also verbal abuse, harassment, and exclusion. Consistent with earlier work [14, 24], we find that women and girls who have not undergone FGM/C are often contemptuously labeled “solema,” a powerful invective translating literally as “uncut,” but also meaning rude, ignorant, sexually unrestrained, uncivilized and unclean. Uncircumcised women generally have little opportunity to oppose the circumcision of their own daughters and they are not allowed to attend the ceremonies or even to visit their daughter during the healing process. In many ways these women remain marginalized in their new compounds; some report that excised family members cast insults at uncut women, refuse to eat food that they prepare, and exclude them from family events such as weddings, as well as from general household decision making and serious discussions among mature women.

We interpret much of the teasing and exclusion that uncircumcised girls and women experience as part of circumcised girls and women using their cut status as a powerful marker of identity, and to demark insider status that affords inclusion and access to social support (see also [29]). Such female social pressure manifests itself not only in the context of inter-ethnic marriage, but also in mixed-ethnicity peer groups of girls and young women.

Even the children insult their mates who are not circumcised as “solema.” It is common to hear children calling their fellow children: “You solemas, you will not follow us (hang out with us).”

-Young Mandinka woman, rural Gambia

Parents who choose not to circumcise a girl commonly face judgments about whether they are raising their girl properly, an issue that is understood to influence standing of the entire extended family.

Such a child (a circumcised girl) brings a good reputation to her family members, especially her parents since everyone knows they have raised this girl to behave (properly).

–Older Mandinka woman, rural Gambia

Pressure to conform arose not only from negative sanctions such as exclusion and insults, but also from internalized moral norms. Research has suggested observations of disparaging treatment of other social groups can lead to the development of internalized evaluations of right and wrong, and that may persist when the group is not present [6]. In line with this, we observe that for many women from circumcising families, FGM/C represents an embodiment of moral virtue. The practice is a constitutive element in the construction of identity and personhood that holds meaning beyond simply that of body modification; the physical act of cutting can have a morally transformative value, as virtue and honor become inscribed upon the body. Women who have been cut are described as “smooth,” “clean,” and “pure,” as well as morally superior to uncut women. Conversely, uncut women are frequently described in terms of social difference, condemnation and repulsion: they “smell,” and are “unclean” and scorned. Moreover, we found that even when cut women had come to question the value of FGM/C due to health risks, legal restrictions, or other reasons, they often found it difficult to discard notions of immorality, malodorousness, and repugnance of uncut women, and commonly expressed conflicting views on their feeling about continuation of the practice (see also O’Neil [33]).

When themes related to pressure and exclusion were raised, there was consistently a positive consensus (Table 1). These themes did not arise in all discussion groups, and were raised less in rural Senegal; we interpret this to mean that pressure to conform among both girls and women may be less relevant. Programs aimed at abandonment of FGM/C and/or legal restrictions may have lessened social pressure. Alternatively, it may be the case that in this low prevalence region, women with FGM/C form social ties with uncut women, and hence experience less pressure to conform with the practice. Contrary to predictions in some of the FGM/C literature (e.g. [4]), pressure to conform was not reduced in our urban study site. The topics of moral virtue and aesthetics of cutting were, by contrast, raised in all our focus groups. Thus, regardless of shifting social circumstances and possible alterations in negative sanctions, internalized moral norms appear particularly resistant to change among all women in our study, irrespective of age or location of residence.

### Upholding tradition while negotiating change

In our Senegambian study communities, when discussing the significance of female circumcision, our informants provided a multitude of reasons for the practice that touched on themes related to notions of proper parenting, training on how to display respect, cleanliness, attenuating sexual desire, and easing childbirth. We find, however, that FGM/C is linked first and foremost to the concept of tradition (sub-themes summarized in Table 2).

**Table 2:** Summary matrix of themes related to the overarching theme of tradition.

	Urban Gambia		Rural Gambia		Rural Senegal	
	Younger Women	Older Women	Younger Women	Older Women	Younger Women	Older Women
Honoring tradition of ancestors	+	+	+	+	+	+
Acceptable change	+/-	+	+	+/-	+/-	+/-
Acceptability of abandonment	-	+/-	-	+/-	-	+/-

+ positive consensus.

+/- divergent views.

-negative consensus.

Blank—theme not raised.

Consistent with prior research [14, 24], numerous informants describe the practice as inherited from ancestors, reciting an oft-repeated phrase, “We found it from our grandmothers.” Being from a “circumcising culture” features centrally in the formation of cultural identity, and girls who have been circumcised are, according to many adherents, thought to have been properly raised to respect ancestral “traditions.” There was wide agreement that FGM/C is central to the formation of personhood, social cohesion and cultural identity. The practice of FGM/C was seen to signify sharing values that are considered central to identity, powerfully marking insider and outsider status, and, as earlier research has emphasized, essential for social inclusion and support, particularly amongst networks of women [29]. This resonates with a broader anthropological literature that finds bodily transformations (through hair, clothes, adornment, or in this case, genital cutting) to be simultaneous social transformations [33]. Every change to the body is justified as a valued cultural practice because it signifies and reaffirms one’s social identity.

Although the notion of upholding traditions of ancestors was by far the most consistent and powerful message associated with FGM/C, this concept was not static, but instead was varied and subject to revision. Certain aspects of circumcision and initiation practices have changed dramatically in recent decades, and these changes were still frequent topics of debate. These include the tendency to perform FGM/C individually and in the absence of training and ritual celebration, and at younger and younger ages [24]. Directly questioning tradition remained an extremely sensitive issue, since challenging the wisdom of elders is considered to be disrespectful. Nonetheless, rather than viewing the practice an immutable cultural practice, dialogues explored acceptable avenues of modifying practices. While number of informants explained that “changes in the practice are vital,” debates appeared as to the types of change that can or should be embraced, and the circumstances under which they might be accepted.

Newer debates on acceptable change center on minimizing health risks, namely by medicalizing the practice. The term “medicalization” refers to a wide range of modifications, ranging from “clinicalization” of the practice and having the procedure performed by medical practitioners, to the adoption of prophylactic or hygienic measures, such as the use of anti-tetanus injections, antibiotics, pain control medications, and sterile cutting instruments, such as razors. While many of our informants favored or were open to the possibility to using sterile instruments and what they referred to as “western medicines,” there was deep resistance to clinicalization, despite the fact that male circumcision has become clinicalized in much of Senegambia. This was seen as a potential challenge to the central and authoritative role of traditional circumcisers (*ngasingbas*), who have unique knowledge of the physical and spiritual aspects of what was considered “proper” circumcision. Additionally, in Senegal, where FGM/C has been outlawed, the practice has in some instances been driven underground, and impeded seeking medical care:

The reason we don’t use medicine is that female circumcision is done in secret. We don’t go to the clinic because there are Wollofs, Sereers and some Fulas in clinics who don’t practice it (FGM/C). That is why we are doing it in secret.

-young Fula woman, rural Senegal

Additionally, survey data have shown that the type of cutting (often described as “cut all” or “cut just a little,” sometimes referred to as *sunna*, Arabic for “tradition” or “duty”) is not changing between generations [26], and the idea of “cutting less,” embraced at times elsewhere in Africa, was categorically rejected by those who support FGM/C. The key point of debate surrounding the physical act of cutting centered on whether or not to cut at all. And in a handful of instances families had opted to abandon the practice of FGM/C. However, in many instances we found a great deal of ambivalence about whether FGM/C is still meaningful under current social conditions, influenced by changing local conditions such as new health concerns (particularly HIV), legal bans, as well as local and global campaigns aimed at ending the practice. Debates that were at times contentious centered on whether FGM/C has become an outmoded tradition that is possibly obsolete, understood to be unacceptably dangerous, or impractical in the current social climate of intolerance.

Notably, across all study locations it was younger women who were most conservative regarding change in or abandonment of FGM/C (Table 2). As young women marry and move to their marital home, FGM/C signals “insider” status among women in the extended family, and thereby helps young women access social support. Hence, young married women have much at stake regarding the continuation of FGM/C in terms of social support and acceptance. Older women, by contrast, were more apt to embrace change or debate the necessity of continuing to practice FGM/C. Moreover, it was widely agreed that older women, as custodians of tradition, play a paramount role in passing down cultural knowledge and assuring that traditional values and practices are maintained. Their experience and wisdom were widely recognized, and their role in promoting the spiritual and physical wellbeing of family members was acknowledged and deeply valued. Our findings reveal that despite their roles as guardians of traditions, older women were not uniformly resistant to incorporating new ideas or practices regarding FGM/C. Instead, a number of senior women expressed the need to seek solutions to changing social circumstances. In some instances, this included having come to oppose FGM/C.

### Social hierarchy and “knowing the eye”

In addition to the importance of honoring tradition, a theme that emerged across all focus group discussions centered on the importance of displaying respect to elders as a means of upholding social hierarchy (Table 3). One important mechanism for displaying respect is to signal subservience, commonly described as “knowing the eye.” This term refers to being able to communicate respect for elders through non-verbal signs, and more generally behave in a fashion that is socially refined in comparison to uncut women. Acknowledgement of social hierarchy makes explicit the power structure that permeates in many realms of social life, including the authority to question the practice of FGM/C.

**Table 3:** Summary matrix of themes related to the overarching theme of upholding social hierarchy.

	Urban Gambia		Rural Gambia		Rural Senegal	
	Younger Women	Older Women	Younger Women	Older Women	Younger Women	Older Women
Respect for elders	+	+	+	+	+	+
Training (to “know the eye”)	+	+	+	+	+	+
Authority to challenge FGM/C	-	+/-	-	+/-		+/-

+ positive consensus.

+/- divergent views.

-negative consensus.

Blank—theme not raised.

The training that traditionally accompanied FGM/C strongly emphasized instruction on how to display respect to elders. As Ahmadu explains, “on one hand the pain and hardship that girls undergo was said to harden and prepare them to be strong and self-assertive in their marital homes. On the other hand, female elders also stressed that through initiation young girls are taught the art of subordination to their husbands, their husband’s brothers and, importantly, to their future mothers in-law” ([34], p. 58). Women who “know the eye” have been indoctrinated into the social hierarchy, and know to signal deference to those in higher positions.

Yes, [if you are circumcised,] you will have respect, you will know the eye. And it will make you be independent, because of the teachings you undergo during circumcision you will be able to stay anywhere.

—Older (circumcised) Wollof woman, urban Gambia

Now that FGM/C occurs at early ages and in the absence of seclusion, training on properly displaying respect is now carried out by women in the extended family throughout a girl’s childhood. Given that social order based on hierarchies of power is anchored in filial piety, a key question regarding FGM/C is, who has the authority to challenge the practice? In the past, elders would designate a time for a large circumcision when enough girls of age to be circumcised had accumulated in the community. The community-wide decision to hold a group circumcision would then instigate decision-making at family and individual levels; for example, whether to participate in the group circumcision or to postpone until the next opportunity, or whether to travel or send a girl to a neighboring community to participate in circumcision there.

As large group circumcisions have become less common, decision making regarding when and how to circumcise has shifted to the family, also providing more opportunity to revisit the question of *whether* (rather than *when*) a girl will be circumcised. Additionally, with the increase in inter-ethnic marriages, instances of FGM/C-discordant marriages (one parents’ family practices FGM/C and the other does not) has complicated decision-making. While customarily ethnicity and FGM/C traditions are said to be inherited patrilineally, the reality is much more complex and situationally negotiated. This ambiguity has begun to shift the bedrock of women’s collective identities and moral personhood, sparking familial debates about modifying or ending FGM/C. Commonly numerous members of the extended family—mothers, co-wives, grandmothers, aunts, and fathers—participate in decision making; where there is conflict, these individuals have different degrees of power (to either prevent circumcision or make circumcision happen). Young mothers commonly expressed having limited authority to challenge the decisions of older women:

They could have circumcised her without telling me about it and I would not say anything because it is her father’s tradition and I am just a mother.

–young Mandinka woman, urban Gambia.

Those who oppose FGM/C could bolster their position by eliciting support from others, particularly senior female women who have power and authority within the community and the family, and in particular have a great deal of authority over junior women in their extended family.

Hernlund predicted that because senior women have the biggest stake in the intergenerational hierarchy of women, they would be more likely than younger women to support the perpetuation of FGM/C and to resist those who challenge the practice [14]. Our findings do not support this assertion. In discussions among younger women, particularly in The Gambia, there was agreement that they lack the authority to question norms associated with FGM/C, as it represents an unacceptable challenge of the authority of older women. Additionally, it is younger women who have the most at stake for violating normative expectations regarding FGM/C. They risk losing social acceptability and social support, as well as self-esteem. By contrast, older women commonly debated amongst one another about shifting social circumstances and whether or how traditions, including FGM/C, should be modified to uphold cultural values in light of social change.

### “One blade per girl”: Shifting views on health risks arising from FGM/C

Messaging on health risks associated with FGM/C has formed a cornerstone of international anti-FGM campaign messages for decades. While conducting ethnographic research in The Gambia in the 1990’s, Hernlund documented workshops and media campaigns that aimed to educate Gambians on the harmfulness of FGM/C, drawing information from activist literature that divided the health risks into categories of short-term, long-term and obstetrical risks [14]. Hernlund observed that circumcised Gambian women frequently and sometimes forcefully rejected claims that FGM/C elevates the risk of obstetrical complications, and produced a “credibility gap”–a disjunction between the subjective experiences of circumcised Gambian women and the purported health risks described in the campaign messages [14].

Consistent with this earlier research, we found that many Senegambian women expressed skepticism about the linking FGM/C to complications during childbirth. Women in several discussion groups contended that FGM/C actually facilitates an *easier* delivery, prevents splitting of the clitoris, and mitigates pain, and some suggested that FGM/C is not only harmless, but healthful (Table 4).

Cut women do not suffer from labor and delivery problems. The solemas (uncircumcised women) do. They experience more pain because of the clitoris.

-Young Tilibonka woman, rural Gambian

**Table 4:** Summary matrix of themes related to the overarching theme of health.

	Urban Gambia		Rural Gambia		Rural Senegal	
	Younger Women	Older Women	Younger Women	Older Women	Younger Women	Older Women
FGM/C as healthful or harmless	+	+/-	+	+/-	+	
Health risks, especially new threat of HIV infection		+		+		+/-

+ positive consensus.

+/- divergent views.

-negative consensus.

Blank—theme not raised.

Importantly, many participants emphasized that if circumcision were truly dangerous, their ancestors would not have practiced it (a notion tied to the perceived disrespectfulness of questioning the wisdom of elders).

While many respondents denied that there are any negative health effects attributable to FGM/C, we found that among older Gambian women, this issue had become a subject to debate, most consistently in The Gambia. A motivating factor appears to be the introduction of messaging about FGM/C as a risk factor for contracting HIV/AIDS. Despite the fact that in the years immediately preceding our study the prevalence of HIV was relatively low in Senegal and Gambia, compared to countries in East or South Africa (1%, according to UNAIDS, 2008) [35], the message of HIV risk was deemed credible among many older women. FGM/C was understood to elevate HIV risk via direct transmission when girls are cut sequentially using a shared instrument.

As a new and dreaded disease, the risk of acquiring HIV through circumcision provided a powerful biomedical rationale for reassessing cutting practices. Because HIV transmission represented a novel danger not faced by earlier generations, alterations of cutting practices to mitigate risk were not seen as challenges to the wisdom of ancestors who handed down these traditions. For strong proponents of FGM/C, the risk of contracting HIV was in in some instances the *only* disadvantage cited to be associated with the practice. The proposed solution to allow for continuation of FGM/C was to implement change in the practice—namely, minimize the risk of direct transmission by using “one blade per girl”:

As a traditional woman, this is part of my culture to practice female circumcision. During the time of our mothers, there was nothing like this transferring of infections. The *ngangsingba* could circumcise many people with one blade and you would see no infections transferred. We are living in a generation where transmitted diseases are rampant. So, (the way) to get rid of these infections is to use one blade per person.

—Older Mandinka woman, rural Gambia

For others, the message on HIV risk led to a reassessment of other aspects of the health risk campaign messages, including the previously oft-rejected claims regarding obstetrical risk. Importantly, among older women, this stimulated discussion about whether FGM/C had become an outmoded custom that should possibly be abandoned.

## Discussion

The factorial focus group methodology used in this study provides a powerful means of illuminating the social norms that uphold FGM/C, and the ways in which meanings have at times become contested or rejected. Our research identified four overarching themes: 1) Pressure to conform, arising from negative sanctions such as ostracization of uncut women and girls, as well as internalized moral norms regarding the association between being cut and embodying virtue; 2) Upholding tradition as a means of venerating ancestors and the cultural values they have imparted; 3) Upholding social hierarchy by displaying respect for elders, including those who are senior in an intergenerational hierarchy of women; and 4) shifting beliefs about the healthful vs. harmful nature of FGM/C. The most striking differences in the reappraisal of these norms occurred along the lines of generation rather than region (urban vs. rural) and country of residence (Senegal vs. The Gambia).

Strong value is placed on upholding tradition as a means of promoting social continuity, passing down the values and wisdom of elders, upholding social hierarchy, and shaping cultural identity. At the same time there is an appreciation that elements of tradition must be reassessed and tailored to meet fluctuating needs and shifting social realities. With regard to FGM/C, older women appear to lead this process of re-evaluation. One new factor that has shaped their views is the threat of HIV infection. For decades many Senegambian women rejected health risk messaging centered on obstetrical risks as a reason to stop practicing FGM/C. Many argued instead that FGM/C is actually healthful, facilitating easier delivery, or at a minimum is harmless, offset by the benefits of creating moral female bodies and upholding social hierarchies and tradition. This view remained widespread among younger women who participated in our study. Acknowledgment of the medical harm was, in their view, equated with challenging the wisdom of elders, undercutting the paramount importance of respecting ancestors and the customs they have passed down. Conversations on potential harm have, however, taken a different turn among older women as messaging has linked FGM/C to the transmission of HIV, a new and dreaded risk not faced in earlier generations. This novel biomedical rationale for stopping or altering FGM/C does not inevitably challenge the wisdom of elders nor the social order anchored in filial piety. Thus, in this manner FGM/C has begun to slip out from under the aegis of morality and its centrality in maintaining social continuity and hierarchies of authority. Under debate are new ways to enable social continuity and uphold moral standards in a shifting social context.

Contours of women’s collective identities and moral personhood are shifting, but not evenly across all segments of society. For many girls, women and their families, concerns are expressed over the enforcement of social norms through negative sanctions. Social pressure regarding FGM/C appears to have been less relevant among girls and women in our Senegal study communities; the topics of peer pressure and fear of social exclusion were not raised during focus group discussions. It may be the case that programs aimed at abandonment of FGC and/or legal ban may have shifted norms and alleviated social pressure. Alternatively, it may be the case that in this low prevalence region, women with FGM/C form social ties with uncut women, thereby shifting their reference group and experience less pressure to conform with the practice. Research on social networks is needed to investigate this possibility. Our findings, however, clearly point to the fluidity of meanings and values, resulting in what Hernlund and Shell-Duncan described as “ever-shifting cultural menus from which people construct their cultural repertoires regarding FGM/C” ([36], p. 45). Increasingly scholars have emphasized that if culture is viewed a set of practices and meaning shaped by context and open to change, local norms may be viewed not as an impediment, but instead as resource for change [10, 11, 13]. Vogt and colleagues concur, arguing that “programmes that take… local heterogeneity as a starting point for promoting abandonment offer a promising avenue for cultural change” [16, p.506]. At the same time, however, it is important to pay close attention to the structures of power that influence the dynamics of change. In this respect, our research draws attention to the fact that older women are uniquely positioned to realize the dual goal of honoring tradition while negotiating change.

Aubel and colleagues argue that a series of negative biases regarding the role of older women tend to cause them to be marginalized or excluded from community health promotion programs [37]. Negative stereotypes include the belief that senior women area not amenable to learning new ideas or adopting new practices, and that they invariably promote traditional practices that tend to be harmful. This assertion may be equally applied to FGM/C, which in the transnational anti-FGM discourse has been labeled a “harmful traditional practice.” Recent research in Sudan, however, finds the older women can be positively influenced by FGM/C behavior change programs; after viewing a film that dramatized divergent views on FGM/C, older participants had more positive views toward uncut girls than younger participants [16]. Aubel and colleagues argue that positive influence of older women can be used across collectivist societies with multigenerational childcare systems to promote the uptake of improved child health practices, including but not limited to ending FGM/C [38]. They formed The Grandmother Project to advance methodologies that draw on “grandmothers” (older women) as change agents, drawing on their established lines of authority and roles as advisors on the care of children [37].

In this study we find that the openness of some older women to reassessing norms and practices suggests that they are amenable to change as they seek solutions to maintaining not just the physical well-being, but also the moral integrity and cultural identity of girls in their family. Despite their roles as custodians of tradition, we found numerous instances in which older women’s views on FGM/C show a great deal of fluidity and reflect syncretic approaches to understanding meanings, rationales and practices related to FGM/C in dynamic, ever-changing contexts. In essence, they are assuring continuity in tradition and cultural identity by carefully negotiating change. By contrast, younger women who lack the moral authority to question norms and challenge the custom of elders were much less likely to express ambivalence or opposition to norms surrounding FGM/C. Given the authority of older women over younger women, and their openness to considering change, it would be of great interest to have a deeper understanding of women’s social networks and key channels of influence both within and across communities. The break characteristics in this study were limited to generation and region and country of residence (urban vs. rural, Senegal vs. The Gambia). To add to our understanding of the intersections of ethnicity and class, these should be included as break characteristics in future research. Additionally, future research should investigate gender norms and whether and how men are configured in these networks. Such information may be useful in efforts optimize the effectiveness of programs aimed at accelerating abandonment of FGM/C.

### Implications for interventions

In brief, our findings suggest that among women in our study communities, older women are the *most* open to changes to the tradition of FGM/C. At first glance this may appear to be paradoxical since one of the primary norms perpetuating FGM/C is reverence for tradition, playing an integral role in bolstering the authority of older women over younger women, as well as promoting social continuity and the formation of cultural identity and personhood. A phrase repeated to us many times, “we found it from our grandmothers” is a powerful explanatory rationale for continuation FGM/C. However, while older women can use their power to perpetuate the tradition of FGM/C, they do not do so uncritically. They also showed an ability to appraise shifting social realities and the power to negotiate change. For this reason, we suggest that intervention programs aimed at accelerating abandonment of FGM/C may be strengthened by specifically targeting and utilizing older women as potential change agents within their communities. Additionally, by drawing on information on the heterogeneity in cultural meanings and values, and the ways in which they are being reassessed in light of shifting social realities, it is possible to draw on cultural variability as a tool for guiding action and social transformation. By recognizing the authority of older women as advocates for change, and drawing on variability and fluidity in norms and practices, it may be increasingly possible to shape possibilities for action and accelerate abandonment of FGM/C without undermining the value of cultural traditions.
